# Examining Factors of Accelerometer-Measured Sedentary Time in a Sample of Rural Canadian Children

**DOI:** 10.3390/children7110232

**Published:** 2020-11-17

**Authors:** Brenton L. G. Button, Gina Martin, Andrew F. Clark, Megan Graat, Jason A. Gilliland

**Affiliations:** 1 Human Environments Analysis Laboratory, Western University, London, ON N6A 3K7, Canada; gmarti57@uwo.ca (G.M.); aclark2@uwo.ca (A.F.C.); mgraat@uwo.ca (M.G.); jgillila@uwo.ca (J.A.G.); 2 Department of Geography and Environment, Western University, London, ON N6A 5C2, Canada; 3 Children’s Health Research Institute, London, ON N6A 5A5, Canada; 4 Department of Paediatrics, Western University, London, ON N6A 3K7, Canada; 5 Department of Epidemiology & Biostatistics, Western University, London, ON N6A 5C1, Canada; 6 School of Health Studies, Western University, London, ON N6A 3K7, Canada; 7 Lawson Health Research Institute, London, ON N6C 2R5, Canada

**Keywords:** rural, child, sedentary time, Northern Ontario, weather

## Abstract

The aim of this study was to examine potential child-level and day-level factors of accelerometer-measured sedentary time in a sample of rural Canadian children. Children (*n* = 86) from rural Northwestern Ontario participated in this study. Children’s sedentary times were identified and logged using an accelerometer. Child-level data (socio-demographic, household, and environment) came from surveys of children and their parents and a passively logging global positioning unit. Day-level data on day type (weekday/weekend) and weather (temperature, precipitation) were based on the dates of data collection and meteorological data came from the closest Environment Canada weather station. Cross-classified regression models were used to assess the relationship between child-level and day-level correlates of sedentary time. Boys were less sedentary than girls (b = −30.53 *p* = 0.01). For each one-year age increase, children’s sedentary time increased (b = 12.79 *p* < 0.01). This study indicates a difference in sedentary time based on a child’s age and gender. However, family, environmental, and weather characteristics did not influence sedentary time in this sample. Health practitioners who deliver care for northern rural youth can provide targeted health advice regarding sedentary time and consider gender and age to be risk factors for these behaviors.

## 1. Introduction

In Canada, children are spending too much time sitting and not enough time moving; approximately 50–60% of waking hours are spent in sedentary time [1,2]. Sedentary time refers to any waking behavior characterized by an energy expenditure ≤ 1.5 metabolic equivalents (METs) while in a seated or lying position [3]. For reference, walking on a flat road at 2.7 kph [1.7 mph] is about 2.3 METs [4], while activities below 1.5 METs include sitting, typing, reading, or meditating [5]. This large proportion of time spent in sedentary behaviors is a public health concern, as higher levels of sedentary time have been associated with negative health outcomes including obesity risk markers, lower levels of aerobic fitness [6], and poor cognitive health [7]. These behaviors have been shown to continue into adulthood, indicating that early interventions may improve the health of the general population [8].

There is currently a large gap in rural sedentary time research, as few studies have focused on objectively measured sedentary time in rural Canadian children [9,10]. Rural areas have been increasingly recognized as a space where residents have poorer health outcomes than their urban counterparts [11,12]. Rural residents often have lower incomes, poorer educational profiles, and less access to health and recreational services, which can affect the health of children and adolescents [13]. Research has suggested that factors that influence health behaviors in rural children differ from children living in urban environments [14,15] Therefore, it is crucial that researchers understand the determinants of sedentary time in rural children to ensure sedentary time is not another behavior where inequities exist.

Sedentary time is a complicated behavior that is associated with many child-level variables (socio-demographic, household, and environmental factors). For example, age, gender, parental income, parental education, access to screens, and supportive built environment features have all been related to children’s sedentary time [16]. Research has also suggested a difference in sedentary time based on temporal variables, such as day type (weekday/weekend) or weather-related variables including rain and temperature [16,17].

Taken together, a major limitation in the sedentary time research is a lack of studies that examine relationships between child-level (e.g., socio-demographic, household, and environment) and day-level factors (e.g., weekday/weekend, temperature, and precipitation) on sedentary time in rural children. To address this limitation, this study will answer the following research question: What are the child-level and day-level correlates of accelerometer-measured sedentary time in a sample of rural Canadian children? This research will benefit practitioners and inform policy designed to target interventions that aim to decrease sedentary time among children in a rural setting.

## 2. Materials and Methods

### 2.1. Study Design and Data Collection

Data were collected from the Spatial Temporal Environment and Activity Monitoring (STEAM) project [18]. The current study uses data from three distinct townships and one First Nation reserve in rural Northwestern Ontario. Populations range in size from approximately 300 to 1650 inhabitants [19]. Data were collected during two time periods: Fall and Winter. Fall data collection occurred between the end of September to mid-October in 2016 and the Winter data collection occurred between the end of November to mid-December in 2016. Prior to participation in the study, each school used their social media account to inform parents about upcoming classroom presentations. During the classroom presentation, the study details were explained to all children in grades 4–8 (ages 8–14). After the presentation, children were given a package to take home to their parents, including a letter of information and parental consent form. After the children returned a signed parental consent form and provided their own assent, they were eligible to participate in the study. Almost 70% of all students in grades 4–8 agreed to participate in the study, as 134 of 194 students agreed to participate. Ethics approval was granted by the Western University’s Non-Medical Research Ethics Board (NMREB: 108029), the two local school boards, and done in accordance with the Declaration of Helsinki.

One parent/guardian and the child completed surveys with questions about demographics and health behaviors. These questions were based on other validated or heavily used survey instruments (e.g., The Neighbourhood Environment Walkability Scale, International PA Questionnaire for children, and the Paediatric Quality of Life Measurement Model) [20,21,22,23]. While children were completing the surveys, they were set-up with an Actical^®^ Z accelerometer (Philips Respironics, Murrysville, PA, USA) and a passive-global positioning system (GPS) unit (Visontac VGPS-900 or Columbus V-900) that they were instructed to wear for seven days during each time period. The GPS data were downloaded from the device during the team’s daily visit to the schools. At the conclusion of the study, the data were imported into ArcGIS (Environmental Systems Research Institute, Redlands, CA, USA) a geographic information systems (GIS) software for visual inspection and data cleaning. The children’s GPS data were combined with a database with built-environment variables describing the opportunity structures for PA.

The sample of 134 was reduced based on the following criteria: (a) they met the accelerometer wear-time criteria described in the following section; (b) had complete data on survey items used in the analysis; and c) had a valid home location determined by GPS data. After applying these inclusion criteria, the final sample included 86 eligible children with a total of 628 valid days of data. This sample did not significantly differ from those excluded based on age, gender, or mother’s education.

### 2.2. Dependent Variable: Sedentary Time

The dependent variable used in this study is the daily number of minutes of sedentary time. Sedentary time was measured using an Actical^®^ Z Accelerometer (Philips Respironics, Murrysville, PA, USA), an omni-directional device worn around the hips sitting on either hipbone, and it has acceptable intra and inter instrument reliability [24]. Sedentary time was considered to be wear time less than 100 counts per minute [25]. This cut-point has been used in previous research, including the Canadian Health Measures Survey, which allows for better comparability to national level data [26]. The accelerometers measured activity in 30-s epochs, which is an appropriate epoch length used for this age group [27]. The accelerometer was set to record movements made by each participant in all directions, summed over a one-minute period. A valid day was considered as 600 min of valid wear time, which is longer than some previous studies [28]. Each student had a minimum of at least one valid day in both seasons. Over 95% of the sample had three or more valid days. Non-wear time was defined as at least 60 consecutive minutes of zero counts, with allowance for two minutes of counts between 0 and 100. The children were instructed to wear the accelerometer during all waking hours, except during water-based activities.

### 2.3. Independent Variables

Independent variables were based on the day when the data were collected (day-level variable) or a measure of a characteristic of the child (child-level variables). A full description of each variable with the data source and description can be found in Table 1.

#### 2.3.1. Child-Level

Child-level variables were gathered from the child and parent surveys as well as by linking GPS data indicating the location of a child’s home to administrative data in a GIS. Data from the child survey included age, gender, ethnicity, number of parents in the household, screen-time rules, and whether the child had a screen in their bedroom. From the parent survey, mother’s education was dichotomized as a proxy for socioeconomic status. From the child’s GPS data, a dichotomous variable was calculated based on whether the child lived directly in one of the larger settled communities of Nipigon (population 1642) or Red Rock (population 895) or in the more rural surrounding area.

#### 2.3.2. Day-Level

Day-level variables included day type (weekday or weekend) and weather variables based on day of data collection. Two binary variables were used to measure precipitation: snow (snow vs. no snow) or rain (rain vs. no rain), and maximum temperature was used as a continuous variable measuring the temperature around the time when children have free time to play outside. Weather variables were gathered from Canada’s Environment and Natural Resources Historical Climate Data website.

### 2.4. Statistical Analyses

A cross-classified model was used to examine the difference in children’s daily sedentary time. This model was selected to account for the two independent sets of clusters that daily sedentary time values are nested in. Specifically, daily values of sedentary time are clustered within each child and, at the same time, they are nested within the date when the data were collected. The cross-classified model allows us to account for this complex data structure. These models are becoming more popular in children’s health research [29,30].

A step-wise approach was used to determine a final model. First, a null model provided an estimate of the variance of daily sedentary time values across children and across dates. Second, variables at the child-level were entered into the model. Third, day-level variables were then added to the null model. Finally, both child-level and day-level variables were added to provide an understanding of how the two levels of factors, when adjusted, influence daily values of sedentary time. All data analyses were conducted using SAS 9.4 (SAS Institute Inc., Cary, NC, USA).

## 3. Results

Table 2 presents the descriptive statistics for the continuous variables and frequencies for the categorical variables included in the analysis. The average age was 10.6 years, and there were more girls (60.5%) than boys (39.5%). A similar distribution was seen for ethnicity, with 59.3% of people reporting being Caucasian/white and 40.7% reporting Indigenous or another ethnicity. On average, the children were getting about 445.9 min (7.4 h) of sedentary time per day.

The results from the first sedentary time model containing all the child-level characteristics suggest that gender and age were significantly associated with sedentary time (see Table 3). On average, boys were getting 28.91 fewer daily minutes of sedentary time than girls (*b* = − 28.91 *p =* 0.02), and each one-year age increase was related to 14.37 more minutes of sedentary time (*b* = 14.37 *p* < 0.01).

The second model contained all day-level variables (see Table 3). These results indicate that no variable had a significant relationship with sedentary time. Finally, the results from the third model were fully adjusted for both child- and day-level factors (see Table 3). Like the unadjusted model, boys were less sedentary than girls (*b* = 30.53 *p =* 0.01), and for each one-year age increase, children’s sedentary time increased (*b* = 12.79 *p* < 0.01) and no day-level variables were significant.

## 4. Discussion

The cross-classified model indicated a difference in sedentary time based on a child’s age and gender. However, family, environmental characteristics, weather, and day type did not influence sedentary time in this sample. Health practitioners and programmers who deliver care for northern rural youth can provide targeted health advice regarding sedentary time while considering gender and age as risk factors for this behavior.

Younger children were less sedentary than older children, which is consistent with some previous research [31]. There are several potential reasons for this relationship. For example, as children age, they typically have different school demands, and research has shown an increase in school sedentary time as children age [31]. Additionally, as children age, higher proportions of children own or have access to a cell phone [32]. This increased access to cell phones and the applications that children use on these phones, such as text messaging, social media, or internet browsing, could have a stronger impact in older rural children, as they report not being able to spend time with their friends because of the physical distance in rural environments [33], so they could be using their phones to connect with their peers. To address age-related trends in sedentary time, a potential weekday solution could include the use of stand-up desks in classrooms, as previous studies have shown that they can reduce children’s sitting time [34]. Previous research on rural children has also suggested a want or need for more programmed activities [33]. However, in these rural environments, few locally employed positions involve children’s health promotion. To address this gap, non-traditional groups, such as religious groups or parent champions, need to be approached by district health units or family health teams about organizing activities for children that reduce sedentary time.

A gender gap was found in this research as girls, on average, were accumulating over 30 min more sedentary time than boys. Numerous studies, including studies conducted in rural communities, have shown that boys are more physically active than girls, which could potentially explain girls’ higher levels of sedentary time when compared with boys [30,35]. In fact, a previous study conducted in the same location as the present study found that boys were getting, on average, 26 more minutes of moderate to vigorous physical activity per day [30]. This is an important finding as research suggests that reallocating as little as 10 min of sedentary time with moderate to vigorous physical activity can lead to improved cardio-metabolic risk profiles [36]. This finding, in combination with other research [37,38], exemplifies that health practitioners need to collaborate with rural girls to increase their physical activity behaviors.

This study also had interesting null findings. First, in contrast to other, less contemporary research, the presence of screens in a child’s room had no influence on sedentary time [39,40]. A potential reason for this finding is that screens, in general, have become so ubiquitous that even if a child does not have a screen in their room, they probably have access to a personal screen in their house or can bring the screen into their room [41]. Second, the lack of significant difference detected between sedentary time on weekdays and weekends, and days with or without precipitation, was counter to other research in general populations, as most research shows children are more sedentary on weekends and days with rain [16,42,43]. A possible reason for which no difference existed between weekdays and weekend days and days with or without precipitation is that rural children are isolated in their homes and spend their free time doing sedentary activities regardless of day type and precipitation. This finding reinforces the need for at-home interventions in rural areas. Finally, temperature verged on significance at the 0.05 level, suggesting that it is an important variable for understanding rural children’s sedentary time.

Two long-term changes that could be effective in decreasing sedentary time in all children is an addition to the province wide health and physical education curriculum and a large-scale tax rebate program. Currently, the health and physical education curriculum is dominated by physical activity and movement competencies with very little attention being paid to the importance of sedentary time, [44] even though sedentary time has been recognized as an essential contributor to overall health [45]. Adding sedentary time education to the curriculum could be beneficial in raising awareness of the negative consequences of sedentary time [46]. A potential policy change is creating tax credits for parents in rural communities for at home exercise equipment like trampolines or exergaming equipment [47] as, in rural areas, attendance in organized activities is complicated by lack of or distance to organized programs.

Several limitations of this study should be considered. First, we used a standardized cut-point selected based on previous research using accelerometers [25], but accelerometer limitations include the inability to capture activities such as cycling, swimming, and activities that occurred when the monitor was removed. Second, this study is limited in some of the recommendations it can make as no modifiable factors proved to be significantly related to sedentary time; however, our study does highlight for whom and when targeted interventions may be useful to reduce inequities in sedentary time. Third, mothers in the sample tended to have higher levels of educational attainment than all women in the study area (e.g., 75.6% attained a college/university degree vs. 43.6% for study area) [19]. However, the area level census data available for comparison includes women aged 15 years and older in private households, not just mothers of elementary school children; therefore, the differences are likely smaller than suggested and the potential for bias minimal. Finally, as the results are from three distinct townships and one First Nation reserve in rural Northwestern Ontario, the results are not representative of all the diverse rural areas in Canada.

## 5. Conclusions

Minimal research exists on children’s sedentary time in rural communities in Canada. In comparison with urban communities, rural areas have fewer healthcare groups and health professionals of all types [48]. Rural general practitioners often establish a close-knit relationship with their patients and often care for people from “cradle to grave” [49]. This study identified key factors that influence rural children’s sedentary time. Gender and age had an impact on sedentary time in these rural communities. Since general practitioners are a patient’s main source of reliable healthcare knowledge, it is pertinent that they are aware of at-risk groups to prevent future health-related concerns and, potentially, relay this information to other community groups or parents, building a community’s capacity to create programs to get children moving.

## Figures and Tables

**Table 1:** Variables associated with children’s sedentary time by child- and day-level.

Variable	Source	Description
Child-level		
Socio-demographic		
Gender	Child survey (categorical)	Self-reported gender as boy or girl (boy/girl)
Ethnicity	Child survey (categorical)	Ethnicity coded as either Caucasian or Indigenous and other ethnicity (Caucasian or Indigenous and Visible Minority)
Age	Child survey (continuous)	Age in years
Home and neighbourhood environment		
Maternal education	Parent survey (categorical)	Mother’s education (high school and below/college and above)
Family composition	Child survey (categorical)	Number of parents in the main household (two parents/lone parent)
Screen-time rules	Child survey (categorical)	Self-reported if child had screen-time rules (yes/no)
Screen in bedroom	Child survey (categorical)	Self-reported if child had a screen in their bedroom (yes/no)
Urbanicity	GIS (categorical)	Categorical variable regarding whether the child lives in or outside the two settled communities (rural-small town/rural)
Day-level		
Maximum temperature	Historical weather data (continuous)	Continuous variable data based on maximum temperature from closest weather station
Rain days	Historical weather data (categorical)	Categorical variable based on whether it had rained that day (rain vs. no rain)
Snow days	Historical weather data (categorical)	Categorical variable based on whether it had snowed that day snow (snow/no snow)
Day type	Weekday or weekend day (categorical)	Categorical variable based on whether data were collected during a weekday or weekend day (weekday/weekend)

**Table 2:** Descriptive statistics of the variables of the child participants in the Spatial Temporal Environment and Activity Monitoring (STEAM) study (*n* = 86).

Variable	*n*	%
Child-level		
Socio-demographic		
Gender		
Boy	34	39.5
Girl	52	60.5
Ethnicity		
Caucasian	51	59.3
Indigenous or visible minority	35	40.7
Age, mean (std dev)	10.6	1.4
Home and neighbourhood environment		
Maternal education		
High school or below	21	24.4
College and above	65	75.6
Family composition		
Two-parent household	73	84.9
Lone-parent household	13	15.1
Screen-time rules		
Yes	41	47.7
No	45	52.3
Screen in room		
Yes	17	19.8
No	69	80.2
Urbanicity		
Rural small town	44	51.2
Rural	42	48.8
Day-level		
Maximum temperature mean (std dev)	10	9.5
No rain or snow	16	59.3
Rain days	4	14.8
Snow days	7	25.9
Day type		
Weekday	18	66.7
Weekend	9	33.3
Outcome Variable		
Sedentary Time mean (std dev)	445.9	100.8

std dev: standard deviation.

**Table 3:** The cross-classified model assessing the relationship between a child’s sedentary time and child variables (Model 1), day-level variables (Model 2), and child and day-level variables (Model 3).

Variable	Category	Model 1			Model 2			Model 3		
		Est	SE	*p*-Value	Est	SE	*p-*Value	Est	SE	*p*-Value
Intercept		442.08	22.30		470.11	15.15		462.16	25.48	
Gender (*ref: Girls*)	Boys	−28.91	12.32	**0.02**				−30.53	12.21	**0.01**
Ethnicity (*ref: Caucasian*)	Indigenous and visible minority	1.92	13.06	0.88				3.56	12.87	0.78
Age	Years	14.37	4.19	**<0.01**				12.79	4.21	**<0.01**
Mother’s education (*ref: High school or below*)	College or above	14.64	10.85	0.18				18.82	10.85	0.08
Family composition *(ref: Two)*	One	10.88	18.11	0.55				3.48	18.28	0.85
Screen in room *(ref: No)*	Yes	5.87	15.00	0.70				3.46	14.84	0.82
Screen-time rules *(ref: No)*	Yes	−13.42	12.26	0.27				−11.08	12.12	0.36
Urbanicity (*ref: Rural-small town*)	Rural	4.40	15.35	0.77				7.81	15.23	0.61
Maximum temperature					−1.70	0.87	0.05	−1.65	0.87	0.06
Rain days (*ref: No*)	Yes				−30.96	19.56	0.11	−32.06	19.42	0.10
Snow days (*ref: No*)	Yes				−23.07	18.65	0.22	−19.60	18.62	0.29
Day type (*ref: Weekday)*	Weekend day				−2.80	13.77	0.84	−1.56	13.67	0.91

Italics indicate reference group. Values in boldface indicate statistical significance ***p***-value < 0.05. Est: estimate, SE: standard error.
